# Do α-acyloxy and α-alkoxycarbonyloxy radicals fragment to form acyl and alkoxycarbonyl radicals?

**DOI:** 10.1186/1860-5397-2-10

**Published:** 2006-05-25

**Authors:** Dennis P Curran, Tiffany R Turner

**Affiliations:** 1 Department of Chemistry, University of Pittsburgh, 219 Parkman Ave., Pittsburgh, PA 15260 USA

## Abstract

The generation of α-acyloxy and α-alkoxycarbonyloxy radicals under reductive conditions in fragmentable probe experiments does not provide unequivocal evidence for the fragmentation of such radicals to give ketones and acyl or alkoxycarbonyl radicals. Instead, standard reduction predominates, even at low tin hydride concentrations. Some ketone product is formed in the α-acyloxy substrate at low concentrations, but it is unclear whether this product arises through a slow radical fragmentation process or an inefficient, chain-breaking oxidative process.

## Introduction

Most organic radical reactions occur through a cascade of two or more individual steps [1–2]. Knowledge of the nature and rates of these steps – in other words, the mechanism of the reaction – is of fundamental interest and is also important in synthetic planning. In synthesis, both the generation of the initial radical of the cascade and the removal of the final radical are crucial events [3]. Many useful radical reactions occur through chains that provide a naturally coupled regulation of radical generation and removal. Among the non-chain methods, generation and removal of radicals by oxidation and reduction are important, as is the "persistent radical effect" [4].

**Figure 1:**
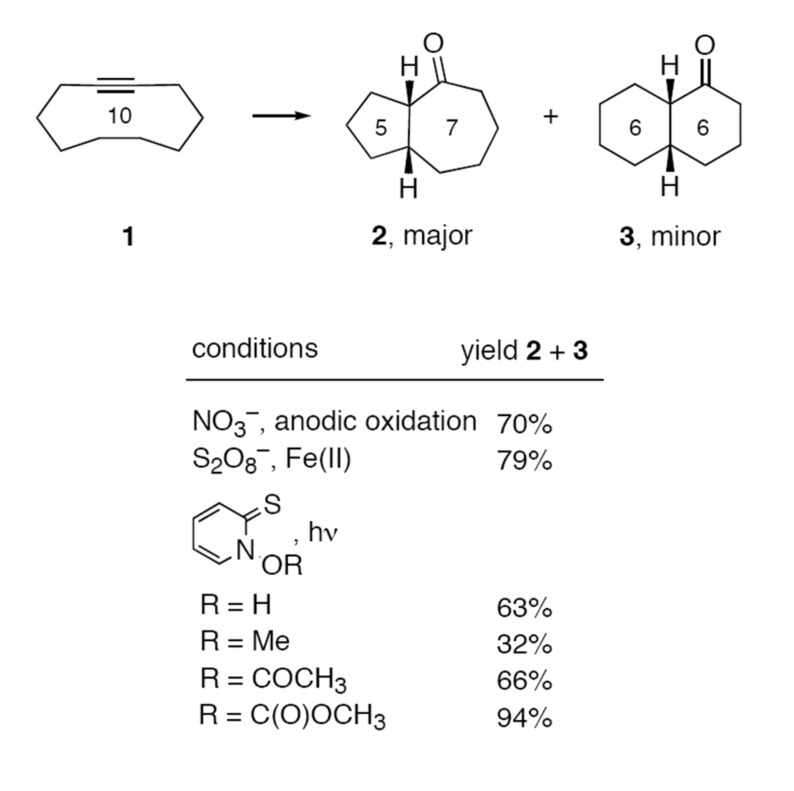
Representative self-terminating radical reactions.

Recently, Wille and coworkers have described a collection of innovative new transformations that they have classed as "self-terminating radical reactions" [5–10]. For example, addition of broad assortment of oxygen-centered radicals to cyclodecyne **1** provides isomeric ketones **2** (major) and **3** (minor) in variable yields, depending on the specific radical involved and the reaction conditions. Representative reagents, reactions conditions and product yields for this very general transformation are shown in Figure 1.

**Figure 2:**
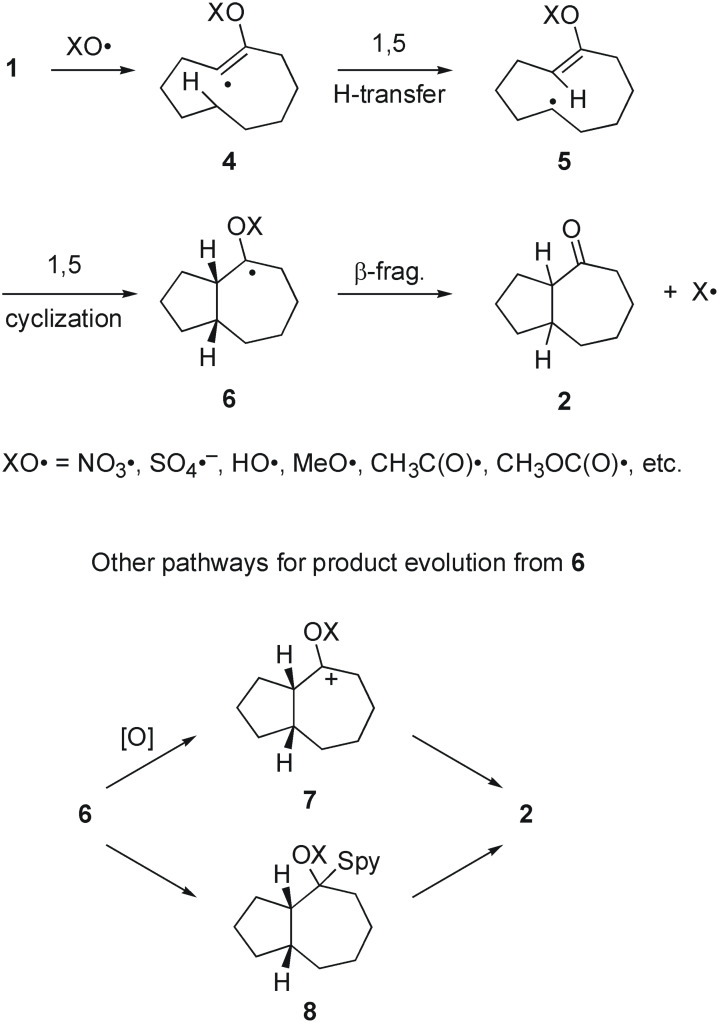
Self-terminating, oxidative and chain mechanisms for evolution of **6** to **2**.

The suggested mechanism for formation of **2** involves addition of an oxygen-centered radical (XO•) to **1** to generate vinyl radical **4**, followed by rapid radical translocation by 1,5-hydrogen atom transfer (Figure 2). The resulting radical **5** rebounds back to the enol ether in a 1,5-cyclization to provide **6**. In the crucial self-terminating step, radical **6** is suggested to fragment to product **2** and radical X•. Related steps are involved in the formation of ketone **3** (not shown), except that the radical translocation occurs by 1,6-hydrogen transfer and the rebound cyclization is 1,6.

The cascade in Figure 2 is a self-terminating, non-chain process if the radical X• does not continue on to propagate a chain in some way. Stable radicals such as X = NO_2_• or SO_3_•^-^ and others are not expected to continue chain propagation. However, other radicals such as X = H•, alkyl (R•), acyl (RCO•) and alkoxycarbonyl (ROCO•) are quite reactive and might be expected to propagate chains under some conditions. Likewise, the stability of radicals X• is also important in the prior β-fragmentation step. If X• is a stable radical such as stannyl, benzyl or *tert*-alkyl, [11–15] then the fragmentation is well precedented [16]. However, for the hydrogen atom and carbon-centered radicals such as methyl and primary alkyl, the fragmentation has little precedent. Recent high level calculations support the notion that related fragmentations to make methyl radicals have high barriers and could be difficult to observe experimentally [17].

Because of the potential difficulties in β-fragmentation of some radicals X•, other pathways for product formation from **6** should be considered. Oxidation (**6 → 7 → 2**) is a relatively common pathway for electron rich radicals like **6** and can even occur under reducing conditions [18–19]. Cation **7** could evolve to ketone **2** by direct loss of X^+^ or through addition of a nucleophile (water or an alcohol, depending on conditions) to give an acetal-type intermediate that would in turn be subject to hydrolysis. In the case of thiohydroxamate precursors, radical **6** could also add back to the initial precursor in the standard Barton "group transfer" mechanism [20]. This would be followed by fragmentation to produce **8** (an acetal form of **2**) and the starting radical XO•. This step begins a new propagation cycle in a chain.

**Figure 3:**
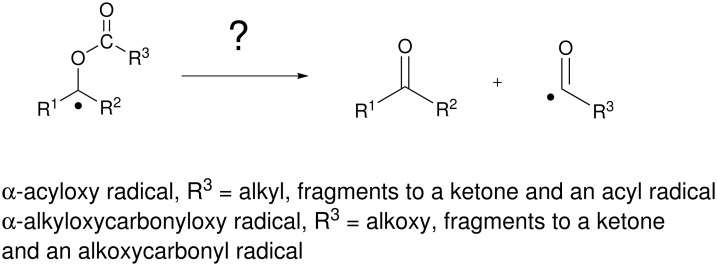
Proposed β-fragmentation reactions to form acyl and alkoxycarbonyl radicals.

We were especially interested in the general β-fragmentation reactions of radicals like **6** to provide either acyl or alkoxycarbonyl radicals (Figure 3). These radicals have many uses in synthesis, [21] so their generation by fragmentation could be a powerful tool. Because acyl and alkoxycarbonyl radicals are stabilized, it is not unreasonable to suggest that such a fragmentation could occur, yet there is nonetheless very little precedent [22–23].

Are the fragmentation reactions in Figure 3 possible, and if so, then how fast are they? If not, then how does Wille's reaction work in such cases? To address these questions, we divorced the other steps of the Wille cascade to isolate the fragmentation reaction for a standard competition kinetic study [24–25]. The results of this study suggest that such fragmentations are very slow reactions at best. In turn, this leads us to suggest that some radicals in the Wille cascade progress to products by oxidation or group transfer rather than β-fragmentation.

## Results and Discussion

**Scheme 1:**
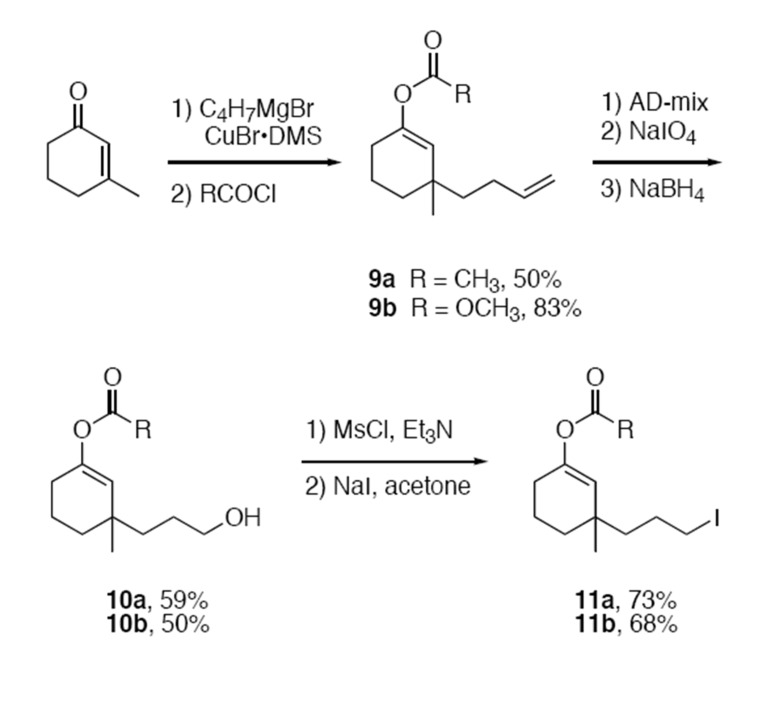
Synthesis of fragmentation probe substrates **11a,b**

We choose to generate the candidate radicals for fragmentation by a radical cyclization rather than by a standard atom or group abstraction reaction because the precursors are readily available and stable (α-halo acetates and carbonates are not stable) and because the intermediate radicals resemble Wille's typical intermediates. The syntheses of precursors **11a** and **11b** are summarized in Scheme 1.

Copper-mediated conjugate addition of 3-butenylmagnesium bromide to 3-methylcyclohexenone followed by quenching with either acetyl chloride [26] or methyl chloroformate provided enol ester **9a** (50%) and enol carbonate **9b** (83%). Oxidative cleavage [27–29] and reduction then provided alcohols **10a** and **10b**, which were converted to iodides **11a** and **11b** through mesylates by a standard procedure. Iodides **11a** and **11b** were stable to heating at 120°C in C_6_D_6_ for 24 h, so polar pathways for product formation are not likely in the cyclization experiments described below.

**Figure 4:**
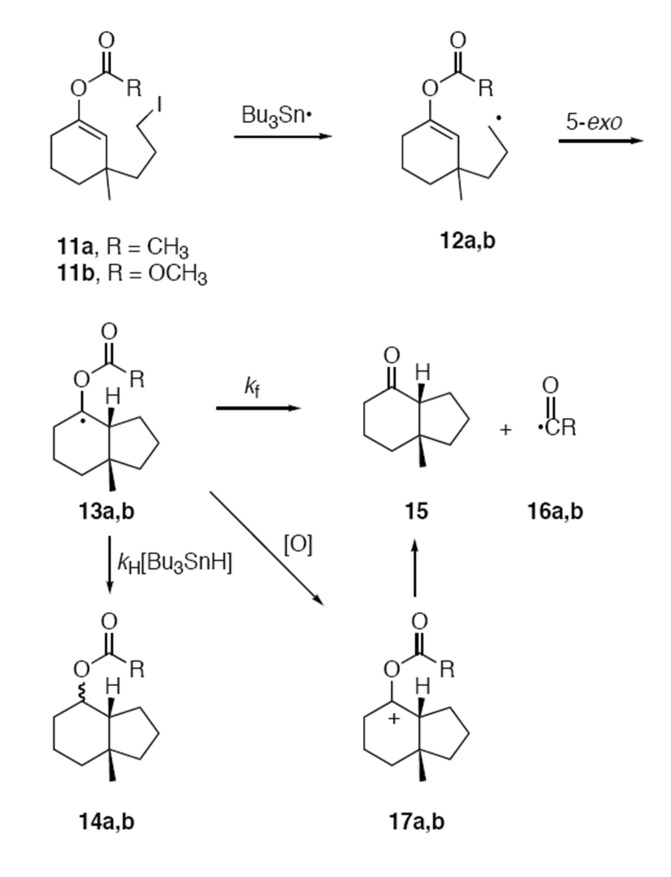
Competing mechanistic pathways for reaction of **11** with Bu_3_SnH.

The projected mechanism for cyclizations of **11a,b** with Bu_3_SnH in a competition kinetics setting is illustrated in Figure 4. Abstraction of iodine from **11** produces alkyl radical **12**, which will rapidly cyclize to give key intermediate α-acyloxy radical **13a** or α-alkoxycarbonyloxy radical **13b**. Partitioning of **13a,b** between bimolecular reduction to give **14a,b** and unimolecular fragmentation to give **15** and **16a,b** is the competition step, and a standard plot of the ratio of products as a function of tin hydride concentration should provide a straight line passing through the origin if radical fragmentation competes with reduction. Alternatively, oxidation of **13a,b** to cation **17a,b** will ultimately also result in the formation of **15**, but the concentration dependence of this process is not clear since the oxidation step is not fully understood.

**Scheme 2:**
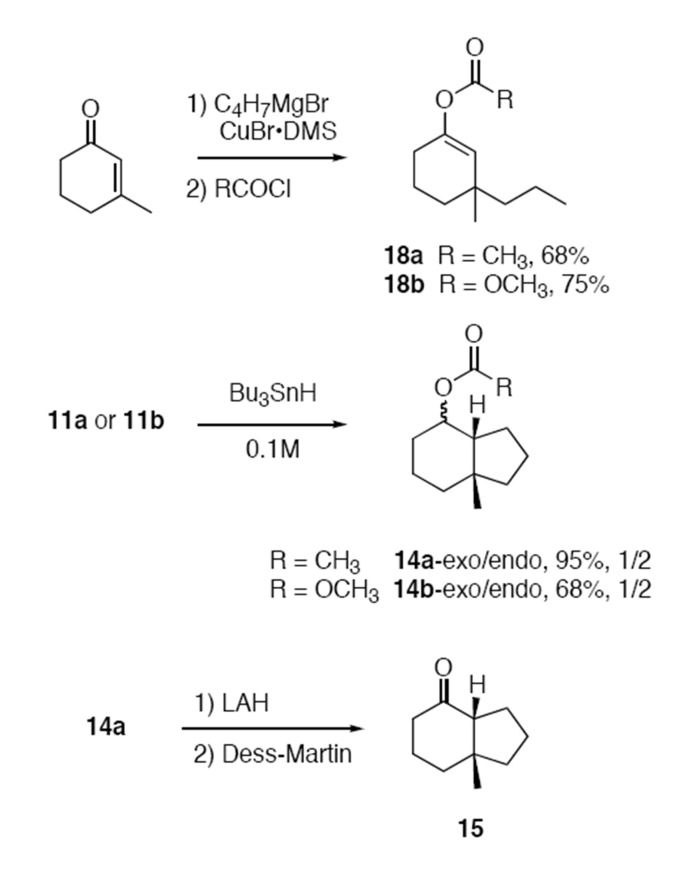
Synthesis of authentic samples of products

Based on the mechanism in Figure 4, authentic samples of all products expected from the cyclizations of **11a** and **11b** were synthesized as shown in Scheme 2. Copper-mediated conjugate addition of propyl magnesium bromide to 3-methylcyclohexenone followed by quenching with acetyl chloride or methyl chloroformate provided reduced, uncyclized products **18a,b**. These products were not detected in any of the subsequent cyclization experiments. Preparative radical cyclization of enol ether **11a** with tributyltin hydride (0.1 M) followed by chromatographic purification provided **14a** in 95% yield as an inseparable 1:2 mixture of *exo* and *endo* isomers. Likewise, cyclization of enol ester **11b** provided a 1:2 mixture of **14b**-*exo* and **14b**-*endo* in 68% isolated yield. The expected product of fragmentation for both substrates, ketone **15**, is a known compound [30–31] that was prepared by reduction of acetate **14a** to provide a mixture of stereoisomeric alcohols (50%), followed by Dess-Martin oxidation (50%) [32].

Competition kinetic reactions were conducted under standard conditions, as detailed in the Supporting Information. Briefly, stock solutions of the iodide **11a,b** (1 equiv) and Bu_3_SnH (1.1 equiv) in C_6_H_6_ or C_6_D_6_ were diluted to the required concentration of tin hydride, then AIBN (0.2 equiv) and *p*-dimethoxybenzene (0.1–0.2 equiv, internal standard) were added. The resulting mixture was rapidly heated to reflux and the progress of the reaction was followed by GC until no further consumption of starting material was observed. Product yields and ratios were then determined by GC and ^1^H NMR analyses. The results of the two analyses were comparable (typically ± 5%), and only the GC results are shown in the Tables. The complete data set is contained in the Supporting Information.

The results of single experiments for the cyclization of enol carbonate **11b** at 0.1 M, 0.01 M and 0.001 M are summarized in the upper part of Table 1 (entries 1–3). At the higher two concentrations, complete conversion of **11b** was observed and reduced product **14b** was formed in good yield. None of the directly reduced product **18b** was observed even at the highest concentration, indicating that the intermediate radical cyclization is fast (*k*_C_ > 10^6^ s^-1^). Negligible amounts of ketone **15** (≤ 2%) were observed, and its yields were not dependent on the tin hydride concentration. Accordingly, no evidence was obtained for fragmentation of intermediate α-alkoxycarbonyloxy radical **13b**. At the lowest tin hydride concentration (entry 3), the conversion stopped with 25% of the starting iodide remaining, but again only a trace of **15** (1%) was detected. These results suggest chain propagation problems at this concentration, which is near the dilution limit for typical radical chain reactions.

**Table 1:** Product Ratios in Bu_3_SnH Mediated Cyclizations of **11a,b**^a^

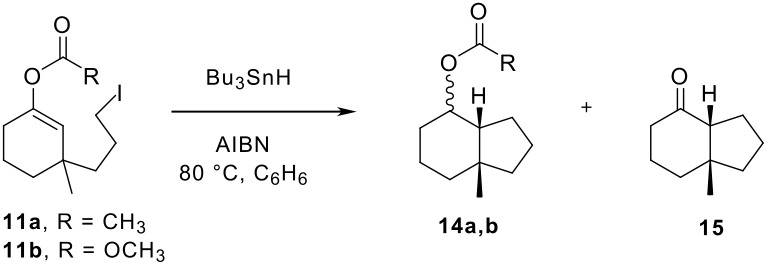						
Entry	Precursor	[Bu_3_SnH]	Yld^b^ **14a,b**	Yld^b^ **15a,b**	Recovered^b^ **13a,b**	Total Yld

1	**11b** ^c^	0.1 M	80%	2%	-	82%
2	**11b** ^c^	0.01 M	70%	1%	-	71%
3	**11b** ^c^	0.001 M	60%	1%	25%	86%
4	**11a** ^d^	0.1 M	95%	2%	-	97%
5	**11a** ^d^	0.01 M	73%	8%	-	81%
6	**11a** ^d^	0.005 M	28%	16%	42%	86%
7	**11a** ^d^	0.001 M	1%	16%	43%	60%

a) C_6_H_6_ or C_6_D_6_, 80°C, b) GC yield against *p*-dimethoxybenzene standard; 2-3/1 mixture of stereoisomers, c) single experiment, d) average of three experiments.

The results for cyclization of enol acetate **11a** at four different concentrations are shown in the lower part of Table 1 (entries 4–7). Since increased amounts of ketone **15** were detected, these reactions were conducted in triplicate, and Table 1 records the averages of the three runs. The raw data in the Supporting Information show satisfactory (± 5% or less) agreement from run to run.

At 0.1 M (entry 4), the reaction of **11a** goes to complete conversion and provides a high yield of reduced product **14a** (95%) along with a trace of ketone **15** (2%). At 0.01 (entry 5), the conversion is again complete and yields of **14a** and **15** are now 73% and 8%, respectively. However, as the reaction is diluted to 0.005 M (entry 6), the conversion of **11a** becomes incomplete (42% recovery), while the yield of **14a** declines to 28% and that of ketone **15** increases to 16%. Finally, at 0.001 M (entry 7), the yield of recovered **11a** is still substantial (43%), while the amount of ketone **15** has stayed the same (16%) and the amount of the cyclized product **14a** dropped to only 1%. A significant amount (40%) of the initial mass balance is unaccounted for in the three experiments at this concentration.

At first glance, the appearance of significant amounts of ketone **15** in the experiments with **11a** at lower concentrations seems to support the fragmentation of radical **13a** to release an acyl radical **16a**. However, the ratios of **15/14a** do not fit well with the standard model of competing unimolecular (fragmentation) and bimolecular (reduction) reactions in Figure 4. For example, the 10-fold dilution in going from entry 4 to entry 5 should have resulted in a **15/14a** ratio about two times higher then was observed. In contrast, the small change in concentration going from entry 6 to 7 now results in an inordinately large increase in this ratio.

We feel that the results in Table 1 with **11a** might be better accommodated by an oxidation pathway for conversion of radical **13a** to ketone **15** via cation **17a**. Since the nature of the oxidant is not known, it is not possible to interpret the concentration dependence of the product ratios. However, the trends of decreased conversions, decreased yields and lost mass balance are not uncommon in such radical oxidation reactions, especially those run under ostensibly reducing conditions [9]. The oxidation step may be inefficient and is almost surely a chain-breaking event. Thus, when the rate of the unspecified oxidation reaction(s) begins to exceed the rate of reduction of radicals **13a** by tin hydride, the whole process begins to break down, so low conversions and yields result.

AIBN has been suggested to be an oxidant in related reactions, [9,33] so we conducted a series of individual cyclizations of **11a** at 0.01 M with increasing amounts of AIBN. The results of these experiments are summarized in Table 2. If AIBN is acting as an oxidant, then the yield of **14a** should decrease and **15** should increase as the concentration of AIBN increases. These trends were not observed. Instead, the yield of **14a** stayed about constant, while the yield of **15** decreased by a small amount. These experiments do not support the active role of AIBN as anything other than a standard radical chain initiator.

**Table 2:** Effect of AIBN Concentration on Product Yields in Reaction of **11a**^a^

Entry	Equiv AIBN	Yld **14a**	Yld **15a**

1	0.25	73%	12%
2	0.50	71%	15%
3	0.75	76%	10%
4	1.0	77%	8%
5	2.0	71%	9%

a) C_6_H_6_, 80°C, 0.01 M Bu_3_SnH (1.1 equiv relative to **11c**).

## Conclusion

In summary, the results with fragmentation probes **11a** and **11b** show the β-fragmentation reactions of α-acyloxy and α-alkoxycarbonyloxy radicals to give ketones and acyl or alkoxycarbonyl radicals (Figure 3 and Figure 4) are, at best, slow reactions. Only traces of ketone **15** were detected in the reduction of **11b** even at very low concentrations, and a conservative upper limit for the fragmentation of this type of radical at 80°C is <10^3^ s^-1^. Small but variable amounts of ketone **15** (7–16%) were produced during cyclizations of **11b**, so the related α-acyloxy radical fragmentations to give acyl radicals could have rate constants as high as 10^3^ – 10^4^ s^-1^. However, the results can also be interpreted through the intermediacy of cationic precursors of ketones produced by radical oxidation, in which case the rate constant for fragmentation is even smaller. Even if the β-fragmentation is occurring by a radical pathway, it is so slow as to have limited synthetic value in radical chain sequences. The sluggishness of these β-fragmentation reactions is surprising, especially give that they produce a strong C=O bond and a stable radical.

In the bigger picture, the results suggest that continued evaluation of the role of β-fragmentation reactions in self-terminating oxidative radical reactions is worthwhile. While the reaction conditions of our probe experiments and prior preparative experiments are very different, the slowness of the β-fragmentations to produce acyl and alkoxycarbonyl radicals suggests that such reactions may not be very competitive under any standard preparative conditions. If fragmentations do not occur to produce acyl and alkoxycarbonyl radicals with reasonable rate constants, then it is unlikely that fragmentations to produce unstable alkyl radicals (for example, CH_3_•) or a hydrogen atom (H•) will occur. A similar conclusion has recently been reached through calculations by Sigmund, Wille, and Schiesser [17]. Either oxidative processes or group transfer reactions may contribute ketone formation in many of these types of reactions.

Oxidative pathways should also be considered when inorganic radicals such as NO_3_• and SO_4_•- are used as promoters. In such cases, the radicals produced on β-fragmentation (NO_2_• and SO_3_•-) are very stable, so the proposed fragmentation is more likely. However, the inorganic conditions are also more strongly oxidizing. So both oxidation and fragmentation pathways are seem reasonable, and further experimentation will be needed to identify which path is preferred as a function of reaction conditions and fragmenting radical in these cases.

The synthetic value of self-terminating oxidative radical reactions is already evident from the pioneering work of Wille, and added value will accrue as we continue to better understand the details of each of the different processes for conducting such reactions.

## Supporting Information

File 1 Complete experimental details and full spectroscopic data for all new compounds; procedures and data for individual competition kinetic experiments and control experiments (7 pages).
