# Supplementary material for: Do α-acyloxy and α-alkoxycarbonyloxy radicals fragment to form acyl and alkoxycarbonyl radicals?
Source: Beilstein J Org Chem. 2006 May 25;2:10. doi: 10.1186/1860-5397-2-10 (PMC1524793; doi:10.1186/1860-5397-2-10)
Supplement: File 1 — Complete experimental details and full spectroscopic data for all new compounds; procedures and data for individual competition kinetic experiments and control experiments (7 pages). [file Beilstein_J_Org_Chem-02-10-s001.doc]

**ADDITIONAL MATERIAL FOR:**

**Do -acyloxy and -alkoxycarbonyloxy radicals fragment to form acyl and alkoxycarbonyl radicals?**

Dennis P. Curran* and Tiffany R. Turner

Address: Department of Chemistry, University of Pittsburgh, 219 Parkman Ave., Pittsburgh, PA 15260 USA

Email: curran@pitt.edu

Contains complete experimental details and full spectroscopic data for all new compounds; procedures and data for individual competition kinetic experiments and control experiments.

**Table of Contents**

General 2

Synthetic procedures 2

Procedures for radical cyclizations with raw data 6

# Experimental

*General:* All reactions were performed under an atmosphere of argon unless the reaction solvent contained water. The reaction times reported are dictated by TLC analysis of the reaction mixture in comparison to the starting material. Reaction solvents were dried either by distillation or passing through an activated alumina column. Methylene chloride was distilled from CaH_2_ and toluene, benzene, diethyl ether and THF were distilled from Na/benzophenone. Solvents dried by activated alumina were done according to Pangborn, A.B.; Giardello, M. A.; Grubbs, R. H.; Rosen, R. K.; Timmers, F. J. *Organometallics*, **1996**, *15*, 1518-1520.

^1^H and ^13^ C NMR spectra were taken on a Bruker models Avance DPX 300 (300 MHz), Avance 300 (300 MHz), Avance DRX 500 (500 MHz), or Avance 600 (600 MHz) NMR spectrometer. Chemical shifts are reported in parts per million (ppm) downfield relative to TMS using the residual solvent proton resonance of CDCl_3_ (7.27 ppm) or central CDCl_3_ carbon peak (77.0 ppm) as an internal standard or C_6_D_6_ (7.15 ppm for ^1^H and 128.0 ppm for ^13^C). In reporting spectral data the format (δ) chemical shift (multiplicity, *J* values in Hz, integration) was used with the following abbreviations: s = singlet, br s = broad singlet, d = doublet, t = triplet, q = quartet, sext = sextet, m = complex multiplet, dd = doublet of doublets, dt = doublet of triplets, dq = doublet of quartets, ddd = doublet of doublets of doublets.

Infrared spectra were taken on a Mattson Genesis Series FTIR using thin film or neat deposition on NaCl plates. Peaks are reported in wavenumbers (cm^-1^). Low and high resolution electron impact mass spectra were obtained on a Micromass Inc, Autospec with an E-B-E geometry. Chemical ionization spectra were taken on the same instrument using methane as the carrier gas. All peaks reported are in units of *m/e*.

Gas chromatograms (GC) were run on an Agilent 6850 Series GC System with an HP-1 Methyl Siloxane column (Agilent 19091Z-413E, Capillary 30.0 m x 320 µm x 0.25µm). The initial temperature of the program was 150 °C with a temperature ramp of 5°C/min up to 250 °C a helium flow of 2 mL/min and 8.68 PSI was applied. *p*-dimethoxybenzene was used as internal standard and C_6_D_6_ or benzene was used as solvent. GC data are reported with a retention time and % area of the total integrated area.

Thin layer chromatography was performed on silica gel 60 F_254_ glass backed plates with a layer thickness of 0.25 mm manufactured by E. Merck. TLC visualization was performed by illumination with a 254 nm UV lamp or by staining with phosphomolybdic acid or permangenate solution and subsequent heating. Flash chromatography was performed on silica gel (230 – 400 mesh ASTM) purchased from Sorbtech or Bodman.

**Acetic acid 3-but-3-enyl-3-methylcyclohex-1-enyl ester (9a).**[[1]](#endnote-2)

Preparation of the Grignard reagent: Magnesium (0.40 g, 16.3 mmol) and a crystal of iodine were placed in a dry three-neck 50 mL round bottom flask attached to a reflux condensor and addition funnel. The contents were flame dried and cooled under argon. 4-Bromo-1-butene (1.38 mL, 13.62 mmol) in dry Et_2_O (20 mL) was added dropwise over 10 min via addition funnel and the mixture was refluxed for an additional 10-15 min and then cooled.

To a dry three-neck 125 mL round bottom flask, attached to a reflux condensor and addition funnel, was added CuBr•DMS (0.19 g, 0.91 mmol), 3-methylcyclo-hexanane (1.03 mL, 9.08 mmol) and dry DMS/ether (40 mL, 50:50) under argon. The solution was cooled to 0 °C and the Grignard reagent was transferred via cannula to the addition funnel and added dropwise over 1 h. The mixture was warmed to RT after addition for 1 h and then recooled to 0 °C. Acetyl chloride (3.20 mL, 45.4 mmol) was added and the mixture was then allowed to stir at RT overnight under argon. The reaction mixture was quenched with sat’d. NH_4_Cl (10 mL) and the layers were separated. The aqueous layer was extracted with Et_2_O (20 mL), the combined organic layers were washed with sat’d. NH_4_Cl (4 x 20 mL), dried over MgSO_4_ and concentrated in vacuo. The crude mixture was purified by column chromatography (98:2 Hexanes:EtOAc) to give **9a** (0.95 g) as clear oil in 50% yield. ^1^H NMR (300 MHz, CDCl_3_) δ 5.78 (dddd, *J =* 17.3, 13.1, 10.1, 6.8 Hz, 1H), 5.11 (s, 1H), 4.94 (dd, *J =* 17.3, 1.9 Hz, 1H), 4.90 (dd, *J =* 10.1, 1.9 Hz, 1H), 1.95-2.16 (m, 4H), 2.06 (s, 3H), 1.69-1.77 (m, 2H), 1.30-1.55 (m, 4H), 1.01 (s, 3H); ^13^C NMR (75 MHz, CDCl_3_) δ 168.6, 147.2, 138.8, 122.3, 133.7, 41.6, 34.4, 33.8, 28.2, 27.0, 26.6, 20.6, 19.1; IR (neat) 1755, 1686, 1363 cm^-1^; LRMS (EI) (M – CH_3_) 193, 151. 111, 84 *m/e*; HRMS (EI) cal’d for 193.1221, found 193.1221.

Acetic acid 3-methyl-3-(3-oxopropyl)cyclohex-1-enyl ester.**[[2]](#endnote-3)**

To a 50 mL round bottom flask equipped with a stirrer was added H_2_O (10 mL), *t*-butanol (10 mL), and AD mix-α (3.36 g) and the mixture stirred vigorously at RT for 0.5 h until 2 clear layers were formed. The mixture was cooled to 0 °C and **9a** (0.50 g, 2.23 mmol) was added neat and the mixture was stirred at RT overnight. Solid sodium sulfite (3.60 g) was added to the mixture and stirred for an additional 30 min. The suspension was diluted with DCM (25 mL) and layers separated. The aqueous layer was extracted with DCM (3 x 15 mL), dried over MgSO_4_ and concentrated in vacuo to give the diol as a clear yellow oil that was used in the next step without further purification. **Diol:** ^1^H NMR (300 MHz, CDCl_3_) δ 5.07 (s, 1H), 3.56-3.59 (m, 2H), 3.37-3.41 (m, 1H), 2.9 (bs, 2H), 2.04-2.12 (m, 2H), 2.08 (s, 3H), 1.71-1.75 (m, 2H), 1.26-1.40 (m, 6H), 0.91 (s, 3H).

To a 50 mL round bottom flask was added the diol, NaIO_4_ (0.51 g, 2.36 mmol) and THF/H_2_O (16 mL, 3:1 ratio) and the reaction mixture was stirred at RT overnight. The resulting mixture was poured into H_2_O (10 mL) and extracted with Et_2_O (5 x 20 mL). The organic layers were combined, dried over MgSO_4_ and concentrated in vacuo. The crude mixture was purified by flash chromatography (80:20 Hexanes: EtOAc) to give the aldehyde as a clear oil (0.35 g) in 75% yield over 2 steps. ^1^H NMR (300 MHz, CDCl_3_) δ 9.59 (s, 1H), 4.88 (s, 1H), 2.29 (t, *J* = 7.8 Hz, 1H), 1.79-2.00 (m, 2H), 1.91 (s, 3H), 1.45-1.62 (m, 4H), 1.28-1.39 (m, 2H), 0.85 (s, 3H); ^13^C NMR (75 MHz, CDCl_3_) δ 201.9, 168.6, 147.8, 121.4, 38.9, 34.0, 33.6, 33.3, 27.0, 26.4, 20.5, 18.9.

**Acetic acid 3-(3-hydroxypropyl)-3-methylcyclohex-1-enyl ester (10a).**[[3]](#endnote-4)

To a stirred solution of the aldehyde (0.40 g, 1.77 mmol) in MeOH (2.5 mL) at 0 °C was added NaBH_4_ (63.0 mg, 1.68 mmol) portionwise. The mixture was allowed to stir under argon for 1 h at 0 °C and then diluted with H_2_O (6 mL) and extracted with DCM (4 x 5 mL). The organic layers were combined, dried over MgSO_4_, and concentrated in vacuo. The crude mixture was chromatographed (80:20 Hexanes:EtOAc) to give alcohol **10a** as a clear oil (314 mg, 78% yield). ^1^H NMR (300 MHz, CDCl_3_) δ 5.02 (s, 1H), 3.48 (t, *J =* 6.1 Hz, 2H), 2.58 (bs, 1H), 2.00 (s, 3H), 1.90-2.02 (m, 2H), 1.65 (q, *J =* 5.8 Hz, 2H), 1.22-1.48 (m, 6H), 0.92 (s, 3H); ^13^C NMR (75 MHz, CDCl_3_) δ 169.3, 147.2, 122.7, 66.0, 38.4, 34.3, 33.8, 27.2, 27.1, 26.6, 20.9, 19.2; IR (neat) 3368, 1754 cm^-1^; LRMS (EI) (M – C_2_H_2_O) 170, 153, 137, 111 *m/e*; HRMS (EI) cal’d for C_10_H_18_O_2_ 170.1306, found 170.1300.

**Acetic acid 3-(3-iodopropyl)-3-methylcyclohex-1-enyl este**r **(11a).**

To a solution of alcohol **10a** (540 mg, 2.55 mmol) and Et_3_N (0.54 mL, 3.88 mmol) in DCM (10 mL) at 0 °C was added mesyl chloride (0.26 mL, 3.28 mmol). The solution was allowed to stir at 0 °C under argon for 3 h then poured into a mixture of H_2_O (5 mL) and Et_2_O (12 mL). The aqueous layer was separated and extracted with Et_2_O (3 x 12 mL). The organic layers were combined and washed with H_2_O (10 mL), brine (10 mL) and then dried over MgSO_4_ and concentrated in vacuo to give the mesylate as a yellow oil. The crude mesylate (740 mg) was used in the following step without further purification. **Mesylate:** ^1^H NMR (300 MHz, CDCl_3_) δ 5.01 (s, 1H), 4.10 (t, *J =* 6.5 Hz, 2H), 2.91 (s, 3H), 1.96-2.03 (m, 2H), 2.00 (s, 3H), 1.61-1.68 (m, 4H), 1.26-1.41 (m, 4H), 0.93 (s, 3H); ^13^C NMR (75 MHz, CDCl_3_) δ 168.7, 147.5, 121.8, 59.9, 37.8, 36.7, 33.4, 26.9, 26.4, 23.7, 20.6, 18.9, 13.8.

To a solution of mesylate in acetone (36 mL) was added NaI (384 mg, 2.56 mmol) and the mixture was allowed to reflux under argon for 2.5 h. The mixture was cooled to RT and the acetone was evaporated *in vacuo*. The solid mixture was dissolved in H_2_O (10 mL) and extracted with DCM (3 x 10 mL). The organic layers were combined and dried over MgSO_4_ and concentrated *in vacuo*. Chromatography (90:10 Hexanes:EtOAc) gave iodide **11a** (593 mg, 73% yield 2 steps). ^1^H NMR (300 MHz, C_6_D_6_) δ 5.01 (s, 1H), 4.10 (t, *J =* 6.5 Hz, 2H), 2.91 (s, 3H), 1.96-2.03 (m, 2H), 2.00 (s, 3H), 1.61-1.68 (m, 4H), 1.26-1.41 (m, 4H), 0.93 (s, 3H); ^13^C NMR (75 MHz, C_6_D_6_) δ 168.4, 148.2, 122.5, 43.5, 34.5, 34.1, 28.8, 27.4, 27.2, 20.6, 19.6, 7.38; IR (neat) 1754, 1218 cm^-1^.

Carbonic acid 3-but-3-enyl-3-methylcyclohex-1-enyl ester methyl ester (9b).

Carbonate **9b** was prepared in the same manner as acetate **9a** using methyl chloroformate (3.51 mL, 45.4 mmol). The crude mixture was purified by column chromatography (98:2 Hexanes:EtOAc) to give 1.2 g of the carbonate in 58% yield. ^1^H NMR (300 MHz, CDCl_3_) δ 5.84 (dddd, *J =* 16.5, 13.5, 10.5, 6.8 Hz, 1H), 5.23 (s, 1H), 5.05 (dd, *J =* 16.5, 1.5 Hz, 1H), 4.95 (dd, *J =* 10.5, 1.5 Hz, 1H), 3.82 (s, 3H), 2.15-2.21 (m, 2H), 2.03-2.11 (m, 2H), 1.75-1.85 (m, 2H), 1.36-1.61 (m, 4H), 1.06 (s, 3H); ^13^C NMR (75 MHz, CDCl_3_) δ 154.1, 147.8, 139.2, 122.8, 114.1, 54.8, 41.8, 34.8, 34.0, 28.6, 27.2, 26.5, 19.2; IR (neat) 1759, 1441 cm^-1^; LRMS (EI) 224, 169, 125, 84 *m/e*; HRMS (EI) cal’d for C_13_H_20_O_3_ 224.1412, found 224.1411.

**Carbonic acid methyl ester 3-methyl-3-(3-oxopropyl)cyclohex-1-enyl ester.**

This aldehyde (0.73 g) was prepared in 72% yield over 2 steps in the same manner as the prior aldehyde using carbonate **9b** (1.0 g, 4.46 mmol). **Diol:** ^1^H NMR (300 MHz, CDCl_3_) δ 5.16 (s, 1H), 3.74 (s, 3H), 3.53-3.57 (m, 2H), 3.32-3.38 (m, 1H), 2.06-2.10 (m, 2H), 1.68-1.71 (m, 2H), 1.26-1.47 (m, 6H), 0.95 (s, 3H). **Aldehyde:** ^1^H NMR (300 MHz, CDCl_3_) δ 9.76 (s, 1H), 5.17 (s, 1H), 3.78 (s, 3H), 2.40-2.46 (m, 2H), 2.11-2.14 (m, 2H), 1.73-1.77 (m, 2H), 1.62-1.67 (m, 2H), 1.38-1.42 (m, 2H), 1.02 (s, 3H); ^13^C NMR (75 MHz, CDCl_3_) δ 201.6, 153.3, 147.9, 121.2, 54.2, 38.6, 33.8, 33.4, 33.0, 26.6, 25.8, 18.7.

**Carbonic acid 3-(3-hydroxypropyl)-3-methylcyclohex-1-enyl ester methyl ester (10b).**

Alcohol **10b** (508 mg, 69 % yield) was prepared in the same manner as alcohol **10a** from the above aldehyde (0.73 g, 3.23 mmol). ^1^H NMR (300 MHz, CDCl_3_) δ 5.02 (s, 1H), 3.56 (s, 3H), 3.34 (t, *J =* 6.3 Hz, 2H), 1.91-1.94 (m, 2H), 1.53-1.57 (m, 2H), 1.11-1.35 (m, 6H), 0.80 (s, 3H); ^13^C NMR (75 MHz, CDCl_3_) δ 153.6, 147.1, 122.4, 62.4, 54.3, 38.1, 34.0, 33.4, 26.8, 26.6, 25.9, 18.8; IR (neat) 3345, 2938, 1441 cm^-1^; LRMS (EI) (M – CH_3_) 213, 195, 169, 125 *m/e*; HRMS (EI) cal’d for C_11_H_17_O_4_ 213.1268, found 213.1128.

**Carbonic acid 3-(3-iodopropyl)-3-methylcyclohex-1-enylester methyl ester (11b).**

Iodo **11b** (739 mg) was prepared in 68% yield in the same manner as **11a** using alcohol **10b** (585 mg, 2.56 mmol). **Mesylate:** ^1^H NMR (300 MHz, CDCl_3_) δ 5.05 (s, 1H), 4.03 (t, *J =* 6.4 Hz, 2H), 3.61 (s, 3H), 2.86 (s, 3H), 1.94-1.99 (m, 2H), 1.55-1.61 (m, 4H), 1.19-1.34 (m, 4H), 0.86 (s, 3H); ^13^C NMR (75 MHz, CDCl_3_) δ 153.4, 147.7, 121.7, 70.3, 54.3, 37.6, 36.6, 34.0, 33.2, 26.7, 25.9, 23.6, 18.8. **11b:** ^1^H NMR (300 MHz, CDCl_3_) δ 5.02 (s, 1H), 3.56 (s, 3H), 3.34 (t, *J =* 6.3 Hz, 2H), 1.91-1.94 (m, 2H), 1.53-1.57 (m, 2H), 1.11-1.35 (m, 6H), 0.80 (s, 3H); ^13^C NMR (75 MHz, C_6_D_6_) δ 153.8, 148.7, 123.0, 55.6, 44.1, 35.0, 34.7, 29.3, 27.9, 27.7, 19.9, 8.0; IR (neat) 1750, 1220 cm^-1^.

**(*endo* and *exo*) Acetic acid 7-methyloctahydroinden-4-(S)-yl ester (14a).**

Iodide **11a** (1.06 g, 3.13 mmol mmol) was added to a sealed tube equipped with magnetic stir bar and diluted with benzene to 34 mL. AIBN (100 mg, 0.06 mmol) was added to the solution followed by Bu_3_SnH (0.99 mL, 3.44 mmol) via syringe and placed in a preheated oil bath and allowed to stir at 80 °C for 2 h. The reaction was cooled to RT then the benzene was removed in vacuo. Chromatography (100% Hexanes followed by gradient 5-10% Et_2_O) of the crude mixture gave a 584 mg mixture of inseparable diastereomers (1.5:1) **14a** 95% combined yield. **14a-endo:** ^1^H NMR (300 MHz, CDCl_3_) δ 5.00 (dt, *J* = 10.9, 4.8 Hz, 1H), 1.98 (s, 3H), 1.24-1.86 (m, 12H), 1.03 (s, 3H) ); ^13^C NMR (75 MHz, CDCl_3_) δ 170.6, 72.9, 47.9, 42.8, 41.3, 31.5, 25.7, 24.8, 24.0, 21.3, 20.8, 20.3; IR (neat) 1736.7, 1245.8 cm^-1^; GC-MS last eluting (M-OAc) 136, 121 *m/e*. **14a-exo**: ^1^H NMR (300 MHz, CDCl_3_) δ 4.60 (dt, *J* = 11.9, 4.8 Hz, 1H), 2.00 (s, 3H), 1.24-1.86 (m, 12H), 0.98 (s, 3H); ^13^C NMR (75 MHz, CDCl_3_) δ 170.8, 73.9, 49.7, 42.2, 41.4, 36.5, 34.1, 29.2, 28.3, 27.1, 20.7, 19.1; IR (neat) 1736, 1245 cm^-1^; GC – MS first eluting (M – OAc) 136, 121 *m/e*.

**(*endo* and *exo*)-Carbonic acid methyl ester 7-methyloctahydroinden-4S-yl ester (14b).**

Diastereomers **14b** (2:1) were prepared in 68% yield (422 mg) in the same manner as **14a** using iodide **11b** (723 mg, 2.14 mmol). **14b-endo**: ^1^H NMR (300 MHz, CDCl_3_) δ 4.89 (dt, *J* = 11.0, 5.2 Hz, 1H), 3.77 (s, 3H), 1.26-1.87 (m, 12H), 1.06 (s, 3H); ^13^C NMR (75 MHz, CDCl_3_) δ 155.7, 78.4, 54.4, 48.0, 43.1, 41.5, 31.4, 25.7, 24.7, 23.9, 20.8, 20.4; IR (neat) 1747.1 cm^-1^; GC-MS last eluting (M – C_2_H_3_O_3_) 136, 121 *m/e*.

**14b-exo**: ^1^H NMR (300 MHz, CDCl_3_) δ 4.80 (m, 1H), 3.76 (s, 3H), 1.26-1.87 (m, 12H), 1.00 (s, 3H); ^13^C NMR (75 MHz, CDCl_3_) δ 155.4, 78.4, 54.4, 49.7, 42.3, 36.5, 33.7, 29.2, 28.3, 27.1, 20.5, 19.0; IR (neat) 1747.1 cm^-1^; GC – MS first eluting (M – C_2_H_3_O_3_) 136, 121 *m/e*.

Acetic acid 3-methyl-3-propylcyclohex-1-enyl ester (18a).

Acetate **18a** was made in the same manner as **11a** using 1-bromopropane when preparing the Grignard reagent. It was isolated in 68% yield as a yellow oil. ^1^H NMR (300 MHz, CDCl_3_) δ 5.06 (s, 1H), 1.98-2.08 (m, 2H), 2.02 (s, 3H), 1.64-1.71 (m, 2H), 1.39-1.46 (m, 2H), 1.15-1.29 (m, 4H), 0.92 (s, 3H), 0.79-.084 (m, 3H); ^13^C NMR (75 MHz, CDCl_3_) δ 169.2, 147.1, 122.9, 45.1, 34.6, 34.0, 27.1, 26.7, 20.9, 19.3, 17.1, 14.7; IR (neat) 1760 cm^-1^; LRMS (EI) (M – CH_3_) 181 *m/e*.

Carbonic acid methyl ester 3-methyl-3-propylcyclohex-1-enyl ester (18b).

Carbonate **18b** was made in the same manner as **11b** using 1-bromopropane when preparing the Grignard reagent. It was isolated in 75% yield as a yellow oil. ^1^H NMR (300 MHz, CDCl_3_) δ 5.16 (s, 1H), 3.71 (s, 3H), 1.97-2.15 (m, 2H), 1.57-1.84 (m, 2H), 1.36-1.45 (m, 2H), 1.13-1.31 (m, 4H), 0.92 (s, 3H), 0.79-0.83 (m, 3H) ); ^13^C NMR (75 MHz, CDCl_3_) δ 153.9, 147.3, 122.9, 54.5, 45.0, 34.7, 33.9, 26.9, 26.2, 19.2, 17.0, 14.7; IR (neat) 1756 cm^-1^; LRMS (EI) 212 *m/e*.

**(*endo* and *exo*)-7-Methyloctahydroinden-4-(S)-ol.**

To a 10 mL round bottom flask was added a mixture of **14a (**0.50 mmol) in dry Et_2_O (10 mL) and cooled to 0 °C under argon. LAH (0.75 mmol) was added portionwise to the solution and allowed to stir for 30 min. The reaction mixture was quenched with H_2_O (5 mL) and the aqueous layer was extracted with Et_2_O (3 x 5 mL). The organic layers were combined, dried over MgSO_4_ and concentrated in vacuo. Chromatography (80:20 Hexanes:EtOAc) gave a 1.5:1 mixture of alcohols as a clear oil in 50% yield. **endo:** ^1^H NMR (300 MHz, C_6_D_6_) δ 3.68 (dt, *J =* 10.6, 4.6 Hz, 1H), 1.11-1.71 (m, 12H), 0.93 (s, 3H); ^13^C NMR (C_6_D_6_) δ 69.3, 51.6, 41.1, 31.9, 30.2, 25.0, 23.7, 21.5, 20.7, 18.4; IR (neat) 3340.2 cm^-1^; GC-MS last eluting (M-H) 153, 136, 121 *m/e*. **exo:** ^1^H NMR (300 MHz, C_6_D_6_) δ 3.07 (ddd, *J =* 17.6, 9.3, 3.9 Hz, 1H), 1.11-1.71 (m, 12H), 0.90 (s, 3H); ^13^C NMR (C_6_D_6_) δ 71.1, 54.0, 42.6, 35.7, 34.8, 34.1, 29.3, 27.3, 20.9, 20.1; IR (neat) 3340 cm^-1^.

7-Methyloctahydroinden-4-one (15).

To a 1.5:1 mixture of the above alcohol (50 mg, 0.32 mmol) in dry DCM (5 mL) was added Dess-Martin periodane (276 mg, 0.8 mmol) and allowed to stir at RT under argon for 1 h. The reaction was diluted with H_2_O (2 mL) and then extracted with Et_2_O (3 x 10 mL). The organic layers were combined, dried over MgSO_4_ and concentrated *in vacuo* to give 15 in 50% yield. ^1^H NMR (300 MHz, C_6_D_6_) δ 2.00-2.14 (m, 2H), 1.92-1.98 (m, 2H), 1.53-1.60 (m, 2H), 1.38-1.44 (m, 2H), 1.21-1.26 (m, 2H), 0.8 (s, 3H); ^13^C NMR (75 MHz, CDCl_3_) δ 214.0, 60.9, 47.8, 40.4, 39.0, 33.9, 27.4, 27.0, 21.9, 21.4; IR (neat) 1708.3 cm^-1^; LRMS (EI) 151 *m/e*.

## Procedures for Radical Cyclizations

Stock solutions of iodides **11a,b**, and internal standard, *p*-dimethoxybenzene, were made in C_6_D_6_ and kept under argon and frozen when not in use. Aliquots from each solution were taken for a ^1^H NMR and GC sample before and after the allotted reaction time to determine yields. Gas chromatograms (GC) were run on an Agilent 6850 Series GC System with an HP-1 Methyl Siloxane column (Agilent 19091Z-413E, Capillary 30.0 m x 320 µm x 0.25µm). The initial temperature of the program was 150 °C with a temperature ramp of 5 °C/min up to 250 °C a helium flow of 2 mL/min and 8.68 PSI was applied. *p*-dimethoxybenzene was used as internal standard and C_6_D_6_ or benzene was used as solvent. GC data is reported with a retention time and % area of the total integrated area. GC yields were determined by calculating the response factors (RF) of each compound to the internal standard using:

Response factors for each compound is as follows: **14a:** 1.2347; **14b:** 2.8085; **11a:** 1.4241; **11b:** 3.0488; **15:** 13677; **18a:** 1.2691; **18b:** 3.6176.

Retention times for each compound is as follows (min):

**standard:** 3.45; **15:** 4.00; **18a:** 4.55; **14a:** 4.93, 4.82; **18b:** 5.12; **11a:** 7.03; **11b:** 9.40

## Concentration studies

Aliquots of radical precursors **11a-b** (1 equiv) and internal standard, *p*-dimethoxybenzene (0.1 to 0.2 equiv) were added to sealed tubes equipped with magnetic stir bars and diluted with C_6_D_6_ to the proper concentration. AIBN (0.2 equiv) was added to the solutions followed by Bu_3_SnH (1.1 equiv) via syringe and were placed in a preheated oil bath and allowed to stir at 80°C for a predetermined amount of time.

Table 1 Reaction yields of 11a with varying concentrations of Bu_3_SnH

| **[Rxn] (M)** | **Time (h)** | **Vol (mL)** | **Yields** |  |  |
| --- | --- | --- | --- | --- | --- |
| **11a** |  |  | **GC/NMR** | **GC** | **GC/NMR** |
|  |  |  | **14a** | **15** | **11a** |
| 0.1 | 2 | 1.2 | 96.3/97 | 2.2 |  |
| 0.1 | 2 | 1.2 | 92.1/93 | 1.8 |  |
| 0.1 | 2 | 1.2 | 95.9/96 | 3.1 |  |
| **Avg** |  |  | **94.8/95.3** | **2.4** | **0/0** |
| 0.01 | 12 | 12 | 74.9/76 | 6.9 |  |
| 0.01 | 12 | 12 | 74.9/73 | 9.3 |  |
| 0.01 | 12 | 12 | 69.1/70 | 6.2 |  |
| **Avg** |  |  | **73/73** | **7.5** | **0/0** |
| 0.005 | 24 | 30 | 29.4/30 | 17.8 | 41.2/37 |
| 0.005 | 24 | 30 | 28.2/31 | 14 | 39.8/39 |
| 0.005 | 24 | 30 | 27.4/29 | 14.9 | 45.4/40 |
| **Avg** |  |  | **28.3/30** | **15.6** | **42.1/38.7** |
| 0.001 | 24 | 30 | 3.2 | 14.3 | 40.5/40 |
| 0.001 | 24 | 30 | 0 | 17.4 | 46.8/41 |
| 0.001 | 24 | 30 | 0.47 | 15 | 40.9/42 |
| **Avg** |  |  | **1.2/0** | **15.6** | **42.9/41** |

**AIBN Concentration Studies**

Aliquots of iodide **11a** (0.124 mmol) and *p*-dimethoxybenzene (0.05 mmol) were added to sealed tubes equipped with magnetic stir bars and diluted with C_6_D_6_ to 13.6 mL. AIBN (varying eqs.) was added to each solution followed by Bu_3_SnH (0.037 mL, 0.136 mmol) via syringe and were placed in a preheated oil bath and allowed to stir at 80°C for 12 h. AIBN amounts were 0.25 equiv (5 mg), 0.5 equiv (10 mg), 0.75 equiv (15 mg), 1 equiv (20 mg), 2 equiv (40 mg).

Table 2 Reaction yields of 11b with varying concentrations of Bu_3_SnH

| **[Rxn ] (M)** | **Time (h)** | **Vol (mL)** | **Yields** |  |  |
| --- | --- | --- | --- | --- | --- |
| **53b** |  |  | **GC/NMR** | **GC** | **GC/NMR** |
|  |  |  | **14b** | **15** | **11b** |
| 0.1 | 2 | 1.1 | 80/73 | 2 |  |
| 0.01 | 12 | 11 | 70/80 | 1 |  |
| 0.001 | 24 | 33 | 60/55 | 1 | 25/30 |

1. Silvestri MG, et al. *J Org Chem* 1999, **64**, 6597. [↑](#endnote-ref-2)
2. a) Sharpless KB, et al. *J Org Chem* 1992, 2768. b) Kodama M, Shiobara Y, Sumimoto H, Mitani K, Ueno K: *Chem Pharm Bull* 1987, **35**, 4039. [↑](#endnote-ref-3)
3. Areces P, Jimenez JL, de la Cruz Pozo M, Roman E, Serrano JA: *J Chem Soc, Perkin Trans 1* 2001, 754. [↑](#endnote-ref-4)
